# Dose-dependent benefits of iron-magnetic nanoparticle-coated human umbilical-derived mesenchymal stem cell treatment in rat intracranial hemorrhage model

**DOI:** 10.1186/s13287-022-02939-4

**Published:** 2022-06-21

**Authors:** Kuan-Hung Chen, Han-Tan Chai, Kun-Chen Lin, John Y. Chiang, Pei-Hsun Sung, Chih-Hung Chen, Hon-Kan Yip

**Affiliations:** 1 Department of Anesthesiology, Kaohsiung Chang Gung Memorial Hospital and Chang Gung University College of Medicine, Kaohsiung, 83301 Taiwan, ROC; 2 Center for Shockwave Medicine and Tissue Engineering, Kaohsiung Chang Gung Memorial Hospital and Chang Gung University College of Medicine, Kaohsiung, 83301 Taiwan, ROC; 3 Division of Cardiology, Department of Internal Medicine, Kaohsiung Chang Gung Memorial Hospital and Chang Gung University College of Medicine, Kaohsiung, 83301 Taiwan, ROC; 4 Department of Computer Science and Engineering, National Sun Yat-Sen University, Kaohsiung, 80424 Taiwan, ROC; 5 Institute for Translational Research in Biomedicine, Kaohsiung Chang Gung Memorial Hospital, Kaohsiung, 83301 Taiwan, ROC; 6 Divisions of General Medicine, Department of Internal Medicine, Kaohsiung Chang Gung Memorial Hospital and Chang Gung University College of Medicine, Kaohsiung, 83301 Taiwan, ROC; 7 School of Medicine, College of Medicine, Chang Gung University, Taoyuan, Taiwan, ROC; 8 Department of Medical Research, China Medical University Hospital, China Medical University, Taichung, 40402 Taiwan, ROC; 9 Department of Nursing, Asia University, Taichung, 41354 Taiwan, ROC; 10 Division of Cardiology, Department of Internal Medicine, Xiamen Chang Gung Hospital, Xiamen, 361028 Fujian China

**Keywords:** Intracranial hemorrhage, Mesenchymal stem cells, Brain infarct volume, Inflammatory reaction, Neurological impairment

## Abstract

**Background:**

This study tested whether two doses of human umbilical-derived mesenchymal stem cells (hUC-MSCs) were superior to one dose for protecting the brain against intracranial hemorrhage (ICH) induced by intracranial injection collagenase and the capacity of ironic-magnetic-nanoparticles (Ir-MNa) coated hUC-MSCs tracked by MRI.

**Methods and results:**

Adult male SD rats (*n* = 40) were equally categorized into group 1 (sham-operated-control), group 2 (ICH), group 3 [ICH + Ir-MNa-coated hUC-MSCs/1.2 × 10^6^ cells with an extracorporeal magnet over rat head (eCMag)/administered by left internal carotid artery (LICA) at post-3 h ICH], and group 4 (ICH + Ir-MNa-coated hUC-MSCs/1.2 × 10^6^ cells with an eCMag/administered post-3 h ICH by LICA and 24 h by IV) and euthanized by day 28. The result showed that by day 28 after ICH induction the neurological function was severely impaired in group 2 than in group 1 that was significantly improved in group 3 and further significantly improved in group 4, whereas ICH volume exhibited an opposite pattern of neurological impairment among the groups (all *p* < 0.0001). Brain MRI demonstrated that by 4 h after ICH, Ir-MNa-coated hUC-MSCs were abundantly identified in ischemic area in group 4. The protein expressions of inflammatory (TNF-α/MMP-9/IL-1ß/iNOS)/oxidative-stress (NOX-1/NOX-2/oxidized protein)/apoptotic (caspase-3/mitochondrial Bax/PARP)/fibrotic (Smad3/TGF-ß)/mitochondrial-damaged (cytosolic-cytochrome-C) biomarkers displayed an identical pattern of neurological impairment among the groups (all *p* < 0.0001). The cellular expressions of inflammation (CD68+/CD11b+)/brain edema (AQP4+) biomarkers exhibited an identical pattern, whereas the neuronal-myelin (Doublecortin+/NeuN/nestin) biomarkers displayed an opposite pattern of neurological impairment (all *p* < 0.0001).

**Conclusion:**

Two doses of hUC-MSCs were superior to just one dose for protecting the brain against ICH-induced damage and Ir-MNa-coated hUC-MSCs offered a well adopted method for tracking hUC-MSCs homing into the brain.

**Supplementary Information:**

The online version contains supplementary material available at 10.1186/s13287-022-02939-4.

## Background

Strokes are typically divided into two major categories, i.e., ischemic and hemorrhagic [1]. Differing from ischemic stroke caused by interruption of blood supply to brain parenchyma due to arterial occlusion (i.e., atheroembolic stroke), hemorrhagic stroke usually results from the rupture of a blood vessel or a vascular structural abnormality [2]. Despite merely 13-20% of strokes belong to hemorrhagic stroke [3], the mortality of hemorrhagic stroke is much higher than that of atheroembolic stroke [4]. There are two main types of hemorrhagic stroke: cerebral [i.e., also called intracranial hemorrhage (ICH)] and subarachnoid hemorrhage [5, 6]. Undoubtedly, once severe neurologic sequelae develop after cerebrovascular accident, the disability can cause huge social and family economic burdens [7].

Current therapy for hemorrhagic stroke [8, 9] includes medical and surgical management. Medical treatment focuses on the maintenance of stability of airway, breathing, circulation (i.e., control of appropriate systemic blood pressure, intracranial pressure), central body temperature and input/output balance, and prevention of seizure disorder. On the other hand, the purpose of emergent/urgent surgery is to reduce the associated complications of hematoma [10, 11]. Unfortunately, despite current standard method employed, disability is still unacceptably high in hemorrhagic stroke survivors that is serious enough to decrease their employability and social activity [12]. Of importance is that not every victim of hemorrhagic stroke is suitable for or beneficial from surgical intervention in clinical practice [13], suggesting the treatment of hemorrhagic stroke is unmet need. Accordingly, to find a safe and effective way to salvage the patients with severe ICH who are not candidates for neurosurgery is utmost important to patients and physicians.

The pathophysiologic mechanistic basis of ICH caused brain injury [14] are elicited by processes of apoptosis, tissue necrosis, complex innate immune cascade, activated matrix metalloproteinases (MMP), vigorous immune reaction, augmentation of reactive oxygen species (ROS) generation and inflammatory reactions. Accordingly, strategic management that to target against the aforementioned molecular-cellular perturbations may have therapeutic potential for attenuating the ICH-induced neuron death and brain dysfunction and improving neurologic functional integrity.

Plentiful evidence has proved that mesenchymal stem cells (MSCs) can suppress inflammatory reaction and innate and adaptive immunity via ameliorating those of immunogenicity and possess immunomodulatory activity [15, 16]. Additionally, MSCs have a powerful ability for self-renewal by preserving their pluripotency and regeneration of the tissue and organ [17, 18]. Studies have previously further established that allogenic MSC treatment is attractive, safe and promising for those patients who suffered from autoimmune disorders [19–22]. A review from Li et al. [23] showed that hUC-MSC therapy is encouraged as a useful candidate for allogenic cell therapy for ischemic stroke with unique “immunomodulation and immunoprivilege” characteristic, reposition/repairment of the injured neurons, upregulation of endogenous neural cells, secretions of neurotrophic mediators, initiation of vascularization and angiogenesis, inhibition of apoptosis, and downregulation of inflammatory response [24].

Surprisingly, when we reviewed the literature, we found that almost all the preclinical and clinical studies just administered merely one dose rather than two doses of the autologous or allogenic stem cells for treatment of different disease entity. One of the main reasons was to avoid the immune rejection. However, when the treatment strategies of bacterial infection, diabetes mellitus, hypertension or even caners, etc., taken into considerations, we find that continuous/multiple doses of medications are always superior to single dosage for controlling these disease entities. More surprisingly, xenogeneic stem cell therapy is currently rarely reported. These aforementioned issues raise the hypothesis that one dose of the stem cell therapy some time may not be enough for resolving the patient’s disease. Accordingly, we proposed that (1) hUC-MSCs might be a therapeutic option for those patients with severe hemorrhagic stroke and unsuitable for surgical intervention; (2) two doses of hUC-MSCs not only were safe as one-dose counterpart but also more effective than one dose on protecting the brain against ICH. To validate this hypothesis, we performed an animal model of ICH study, followed by hUC-MSCs (i.e., xenogeneic MSCs) therapy.

## Methods

### Ethics

All animal procedures were approved by the Institute of Animal Care and Use Committee at Kaohsiung Chang Gung Memorial Hospital (Affidavit of Approval of Animal Use Protocol No. 2018082702) and performed in accordance with the Guide for the Care and Use of Laboratory Animals.

Animals were housed in an Association for Assessment and Accreditation of Laboratory Animal Care International (AAALAC; Frederick, MD, USA)-approved animal facility in our hospital with controlled temperature and light cycles (24 °C and 12/12 light cycle).

### Procedure and protocol of ICH animal model induction by type IV collagenase proteolytic enzyme (refer to Additional file 1: Fig. S1)

Pathogen-free, adult male Sprague Dawley (SD) rats, 300–320 g (Charles River Technology, BioLASCO Taiwan Co. Ltd., Taiwan) were utilized in the present study. The procedure and protocol were based on our recent report [25]. In detail, the stereotactic apparatus was set up and animals were anesthetized by inhalational 2.0% isoflurane, placed in a prone position on a warming pad at 37 °C for acute ICH induction. To ensure the depth of anesthesia, the rats were tested by the pinch. After loss of consciousness, the rats were fixed on the stereotactic frame using a nose clamp and two ear bars. After shaving the fur, the superior surface of rat head was prepared by sterile iodophor wipes as surgical field. A longitudinal incision was made on the surface of head to expose the bregma. Using the stereotactic apparatus, a left point, 3 mm lateral to the bregma of the coronal suture, was marked. A burr hole through the skull (1 mm) was drilled at the marked point by a high-speed stereotaxic drill. Then, 1.0 μL collagenase type IV (0.125 IU/mL) was then carefully injected into corpus/dorsal striatum (5 mm below the skull) by Hamilton syringe 26 G at a rate of 0.2 μL/min (coordinates: 0.2 mm anterior, 5.5 mm ventral, and 3.5 mm lateral to bregma). The sham control rats only received a needle insertion. The syringe was removed slowly after the injection is completed and sterile bone wax is used to plug the hole quickly. The skin on the surface of head will be closed by using 4–0 monocryl. The rats were removed from the stereotactic apparatus and then allowed to recover in a warmed cage with free access to food and water. The recovery period was about 30 min. Finally, the animals were cared for in a portable animal intensive care unit (ThermoCare®) with food and water for 24 h.

### Assessment the homing of intracarotid administration of Ir-MNa-coated hUC-MSCs in ICH area

**Fig. 1:**
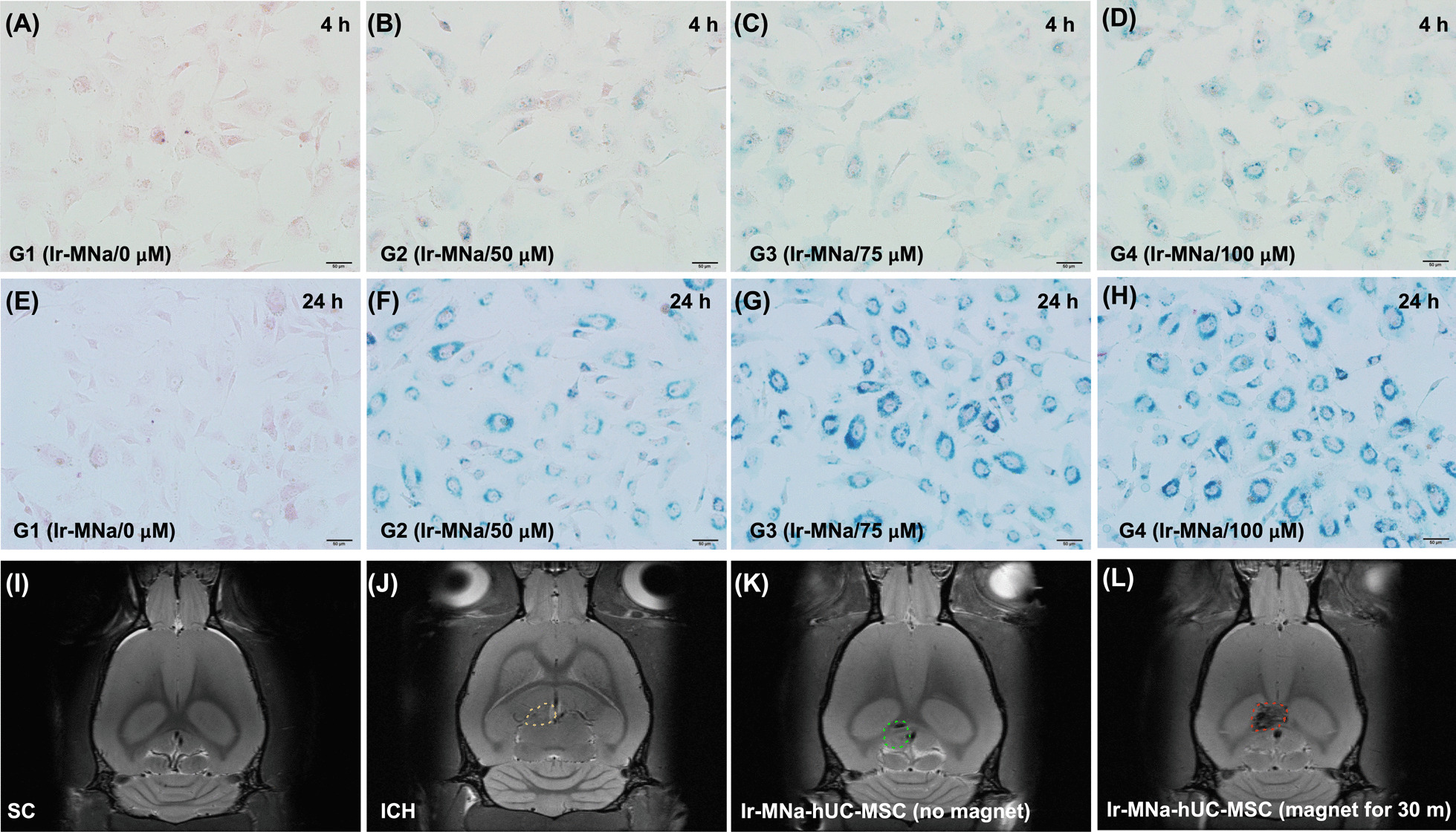
The capacity of Ir-MNa coated in the hUC-MSCs during co-culture and identification of Ir-MNa by brain MRI at 4 h and 24 h after ICH induction. **A–D** at time point of 4 h and **E–H** at time point of 24 h Illustrated the microscopic finding (200 ×). Prussian Blue Stain for identifying the expression of stepwise increased dosages of ironic magnetic nanoparticles (Ir-MNa)-coated in human umbilical cord-derived mesenchymal stem cells (hUC-MSCs) (blue color). The result showed that the Prussian Blue Stain for Ir-MNa in hUC-MSCs demonstrated more stronger expression as the concentration of Ir-MNa was increased. Additionally, the time point of 24 h was better than 4 h for Ir-MNa coating into the hUC-MSCs. Group 1 (G1) = hUC-MSCs coated with Ir-MNa (0 μM); group 2 (G2) = hUC-MSCs coated with Ir-MNa (50 μM); group 3 (G3) = hUC-MSCs coated with Ir-MNa (75 μM); group 4 (G4) = hUC-MSCs coated with Ir-MNa (100 μM). **I–L** Illustrating the brain magnetic resonance imaging (MRI) finding (i.e., for tracking and visualizing cells by MRI) at 4 h after acute ICH induction. As compared with normal (**I**) and ICH (yellow dotted line) + hUC-MSCs without Ir-MNa coating (**J**), the Ir-MNa coated hUC-MSCs (expressed as dark color area) without magnet over the rat head (K) was clearly identified in ICH area (green dotted line). On the other hand, as compared with (K) picture, more spacious dark-color area (in red dotted line area) of Ir-MNa coated hUC-MSCs with magnet over the rat head (L) were observed, suggesting that cells already homing in the hemorrhagic and ischemic regions even at a short time interval (i.e., 4 h) after they were administered. ICH = intracranial hemorrhage; hUC-MSCs = human umbilical cord-derived mesenchymal stem cells

To elucidate whether Ir-MNa-coated hUC-MSCs were early homing into the ischemic and peri-ischemic areas, intracranial injection of collagenase 1μL (0.125U/ml) (Collagenase IV, Sigma-Aldrich, catalog number: C5138) was performed for induction of ICH. For this purpose, the brain MRI was carried out early at 4 h after ICH induction. To perform the brain MRI at this time point was mainly due to the fact that a delayed time interval of this examination would find large amount of blood accumulated in the brain, resulting in rapid accumulation of the iron in the ICH area (i.e., resulted from lysis of red blood cells) that distorted the accurate interpretation of Ir-MNa-coated hUC-MSCs homing to the brain (refer to Fig. 1).

### Animal grouping and the purpose of Ir-MNa utilized in the present study

The study utilized the ironic magnetic nanoparticles (Ir-MNa) coating on the surface of MSCs for cell tracking in living body, i.e., the Ir-MNa served as a contrast agent for precise cell trafficking by MR molecular imaging to assess the final fate of the stem cells.

The animals (*n* = 40) were equally categorized into group 1 (sham-operated control, i.e., SC), group 2 (ICH), group 3 [ICH + Ir-MNa-coated hUC-MSCs (1.2 × 10^6^ cells) with an extracorporeal magnet over the rat head for tracking the cell into the brain/administered by left internal carotid artery (LICA) at 3 h after ICH induction], and group 4 (ICH + Ir-MNa-coated hUC-MSCs (1.2 × 10^6^ cells) also with an extracorporeal magnet over rat head for each time of the cell administration/administered by LICA at 3 h and at 24 h by tail vein with an identical dosage). Animals in each group were euthanized by day 28 after ICH induction and the brain specimen was harvested from each animal for individual study.

### Inclined plane test of hind limb muscle power and coordination (refer to Additional file 1: Fig. S1)

To evaluate the muscle power and coordination of hind limbs of the animals, an inclined plane test (i.e., to measure the muscle tone and stamina) was conducted as we recently described [26]. In detail, during a 3-day period of acclimatization in a temperature- and humidity-controlled room with 12-h light–dark cycle and free access to water and standard animal chow, the rats were gently handled by laboratory personnel five times a day to let them be accustomed to human manipulation. In the following three days of training, the animals were placed on an inclined plane made of cardboard on which a horizontal friction trip provided a foothold for the animal’s hind limbs as the inclination angle increased to prevent the animal from sliding down the slope. During the actual inclined plane test, each animal was placed on the inclined plane so that a secure foothold was established between the claws of its hind limbs and the friction trip. After confirmation of correct body position in the absence of anxious behavior and abnormally tense muscle tone of the animal, the inclination angle was slowly increased till the animal’s hind limbs lost grasp of the friction trip and slid down the plane. The inclination angle was then recorded. After performing the experiment three times for each animal, the mean inclination angle was obtained by averaging the three recordings. The whole procedure was conducted by two independent technicians blinded to grouping of the animals.

### Corner turn test (refer to Additional file 1: Fig. S1)

The procedure and protocol were based on our recent reports [27, 28]. Briefly, each rat was allowed to proceed into a 30-degree corner. To exit the corner, the rat could turn either to the left or right. This procedure was repeated 10–15 times, with at least 30 s between trials, and the percentage of right turns was calculated. Only turns involving full rearing along either wall were included (i.e., ventral tucks or horizontal turns were excluded). The rats were not picked up immediately after each turn to prevent from developing an aversion for their prepotent turning response.

### Procedure and protocol of brain magnetic resonance imaging (MRI) study for measuring the brain hemorrhagic volume (BHV) (refer to Additional file 1: Fig. S1)

The procedure and protocol for brain MRI study were based on our recent report [29]. In detail, the MRI was performed at 16 h and day 28 after acute ICH induction. Briefly, during MRI measurements, rats were anesthetized by 3% inhalational isoflurane with room air and were placed in an MRI-compatible holder (Biospec 94/20, Bruker, Ettingen, Germany). Rectal temperature and respiration were monitored throughout the procedure to ensure normal physiological conditions were maintained. MRI data were collected using a Varian 9.4T animal scanner (Biospec 94/20, Bruker, Ettingen, Germany) with a rat surface array. The MRI protocol was consisted of 40 T2-weighted images. Forty continuous slice locations were imaged with a field-of-view of 30 mm × 30 mm, an acquisition matrix dimension of 256 × 256 and slice thickness of 0.5 mm. The repetition time (TR) and echo time (TE) for each fast spin-echo volume were 4200 ms and 30 ms, respectively. Custom software, ImageJ (1.43i, NIH, USA), was used to process the region of interest (ROI). Planimetric measurement of images from MRI T2 was performed to calculate the stroke volumes of cortex.

### Western blot analysis

The procedure and protocol for Western blot analysis were based on our recent reports [27–29]. Briefly, equal amounts (50 μg) of protein extracts were loaded and separated by SDS-PAGE using acrylamide gradients. After electrophoresis, the separated proteins were transferred electrophoretically to a polyvinylidene difluoride membrane (GE, UK). Nonspecific sites were blocked by incubation of the membrane in blocking buffer [5% nonfat dry milk in T-TBS (TBS containing 0.05% Tween 20)] overnight. The membranes were incubated with the indicated primary antibodies [Caspase 3 (1: 1000, Cell Signaling), Poly (ADP-ribose) polymerase (PARP) (1: 1000, Cell Signaling), mitochondrial Bax (1: 1000, Abcam), tumor necrosis factor (TNF)-α (1: 1000, Cell Signaling), interleukin (IL)-1ß (1: 1000, Cell Signaling), inducible nitric oxide synthase (iNOS) (1: 1000, Abcam), interleukin (IL)-10 (1: 1000, Abcam), NOX-1 (1: 1500, Sigma), NOX-2 (1: 750, Sigma), transforming growth factor (TGF)-ß (1: 3000, Abcam), phosphorylated (p)-Smad3 (1: 1000, Cell Signaling) and Actin (1: 1000, Millipore)] for 1 h at room temperature. Horseradish peroxidase-conjugated anti-rabbit immunoglobulin IgG (1: 2000, Cell Signaling, Danvers, MA, USA) was used as a secondary antibody for one-hour incubation at room temperature. The washing procedure was repeated eight times within one hour. Immunoreactive bands were visualized by enhanced chemiluminescence (ECL; Amersham Biosciences, Amersham, UK) and exposed to Biomax L film (Kodak, Rochester, NY, USA). For the purpose of quantification, ECL signals were digitized using Labwork software (UVP, Waltham, MA, USA).

### Immunofluorescent (IF) staining of brain specimens

The procedure and protocol of IF staining were based on our previous reports [27–29]. In detail, frozen section (4 μm thick) was obtained from the brain hemorrhagic area/at risk area of each animal, permeated with 0.5% Triton X-100, and incubated with antibodies against NueN (1:100, Sigma-Aldrich), nestin (1:200, Abcam), CD68 (1:500, Abcam) and CD11b (1:100, Abcam) at 4 °C overnight. Alexa Fluor 488, Alexa Fluor 568, or Alexa Fluor 594-conjugated goat anti-mouse or rabbit IgG was used to localize signals. Sections were finally counterstained with DAPI and observed with a fluorescent microscope equipped with epifluorescence (Olympus IX-40). Three brain sections were analyzed for each rat. For quantification, three randomly selected high-power fields (HPFs; 400 × for IF study) were analyzed in each section. The mean number of positively stained cells per HPF for each animal was then be determined by summation of all numbers divided by 9.

### Procedure and protocol of Prussian blue staining, flow cytometry and ELISA

To verify the circulating level of Ly6G+, CD11b/c+ or myeloperoxidase (MPO) + cell populations, whole blood cells were stained and performed to flow cytometric analysis (FC500, Beckman) by day 28 after ICH induction. The 1.0 × 10^6^ cells were stained with Ly6g-Alexa Fluor 488 (Abcam), CD11b/c-PE (BD bioscience) or MPO-PE (Abcam) antibodies to detect cell surface markers.

In addition, to analyze circulatory levels of inflammatory biomarkers, serum tumor necrosis factor (TNF)-α and interleukin (IL)-6 concentration were assessed by days 2 and 28 after ICH induction in duplicate with a commercial ELISA kits (R&D Systems).

Furthermore, prior to sacrificing the animals (i.e., by day 28 after ICH induction), the percentages of viable and apoptotic peripheral blood mononuclear cells (PBMNCs) were determined by flow cytometry using double staining with annexin V and propidium iodide (PI) which is a simple and popular method for the identification of apoptotic cells (i.e., early [annexin V+/PI−] and late [annexin V+/PI+] phases of apoptosis).

Finally, to investigate whether the Iron coated in the hUC-MSCs [i.e., ironic-magnetic-nanoparticles (Ir-MNa)- coated hUC-MSCs], the Iron Stain Kit (Prussian Blue Stain) (Abcam, ab150674) was utilized in the present. The procedure and protocol were according to the manufactory instructor.

### Statistical analysis

Quantitative data were expressed as mean ± SD. Statistical analysis was adequately performed by ANOVA, followed by Bonferroni multiple-comparison post hoc test. SAS statistical software for Windows version 8.2 (SAS Institute, Cary, NC, USA) was utilized. A *P* value less than 0.05 was considered statistically significant.

## Results

### The capacity of Ir-MNa coated with the hUC-MSCs during co-culture and identification of Ir-MNa by brain MRI at 4 h after ICH induction (Fig. 1)

To create a reproducible method of Ir-MNa coating in the MSCs to facilitate cell tracking in living body, we first performed an in vitro study. Additionally, to test the Whether stem cells could be coated by Ir-MNa, the optimal administration time point and the suitable dose of Ir-MNa, two-time intervals of co-cultures (i.e., at 4 h and 24 h) and Prussian Blue Stain were utilized. The result of the in vitro study demonstrated that 24 h was better than 4 h for much greater amount of Ir-MNa coated in the hUC-MSCs. Additionally, as for the dose of Ir-MNa (i.e., stepwise increased from, 0, 50, 75 to 100 μM), we found that the 100 μM Ir-MNa was much better than the other lower dose counterparts for coating on the stem cells. Thus, the dose of 100 μM Ir-MNa was utilized for the in vivo study.

Next, we carefully examined the result of brain MRI and identified that the Ir-MNa was clearly observed as a dark color that appeared in the ICH and ischemia-risk areas at a very early time point of 1 h after the cells were administered, suggesting that Ir-MNa-coated hUC-MSCs migrated into the damaged brain regions and was ready for initiating the tissue repairmen and regeneration. Additionally, an extracorporeal magnet over the rat head could accelerate the cells into the brain injured zone.

### The neurological function and the brain hemorrhagic volume (BHV) (Figs. 2, 3)

**Fig. 2:**
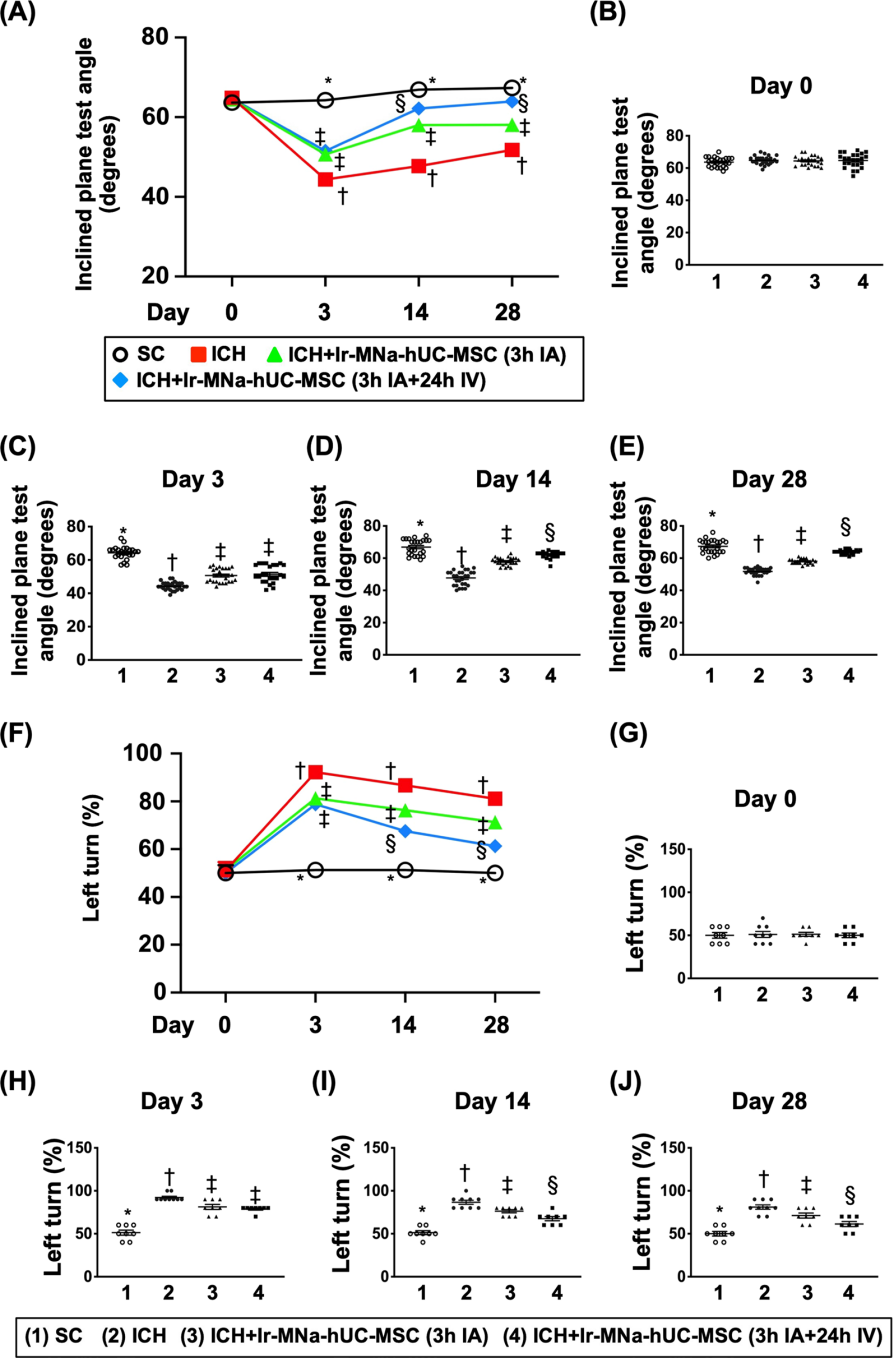
The time courses of neurological function assessment. **A** Illustrating the inclined plane test for determining limb motor function on days 0, 3, 14 and 28 after acute ICH induction. **B** Statistical analysis by day 0, *p* > 0.5. **C** Statistical analysis by day 3, *versus other groups with different symbols (†, ‡), *p* < 0.0001. **D** Statistical analysis by day 14, *versus other groups with different symbols (†, ‡, §), *p* < 0.0001. **E** Statistical analysis by day 28, *versus other groups with different symbols (†, ‡, §), *p* < 0.0001. **F** Illustrating the corner test for determining limb motor function on days 0, 3, 14 and 28 after ICH procedure. **G** Statistical analysis by day 0, *p* > 0.5. **H** Statistical analysis by day 3, *versus other groups with different symbols (†, ‡), *p* < 0.0001. **I** Statistical analysis by day 14, *versus other groups with different symbols (†, ‡, §), *p* < 0.0001. **J** Statistical analysis by day 28, *versus other groups with different symbols (†, ‡, §), *p* < 0.0001. All statistical analyses were performed by one-way ANOVA, followed by Bonferroni multiple comparison post hoc test (*n* = 10 for each group). Symbols [(*, †, ‡, §) indicate significance (at 0.05 level). IA = intra-carotid arterial administration; IV intravenous (i.e., tail vein) administration; SC = sham control; ICH = intracranial hemorrhage; Ir-MNa-hUC-MSC = Ironic Magnetic Nanoparticles/human umbilical cord-derived mesenchymal stem cells

To assess whether the hUC-MSCs treatment would improve the neurological function and reduce the BHV, the corner test, inclined plane test and brain MRI were performed. The inclined plane test (i.e., for determining limb motor function) demonstrated that by day 0, this parameter did not differ among the four groups. However, by day 3 after ICH induction, this parameter was significantly lower in groups 2, 3 and 4 as compared with the SC group (Fig. 2). Additionally, there was a trend of statistical non-significant improvement of neurological function in groups 3 and 4 as compared with group 2 (Fig. 2). By days 14 and 28 after ICH induction, this parameter was highest in group 1, lowest in group 2, and significantly higher in group 4 than in group 3, suggesting that the consecutive administration of two doses of hUC-MSCs might be better than one dose counterpart for improving limb motor function after ICH (Fig. 2).

To further evaluate the recovery of neurological function, we utilized corner test (i.e., sensorimotor functional test) in the present study. The result of corner test again exhibited an opposite pattern of inclined plane test among the four groups, once gain suggesting the consecutive administration of two doses of hUC-MSCs was better than one dose counterpart for improving neurological function after ICH (Fig. 2).

**Fig. 3:**
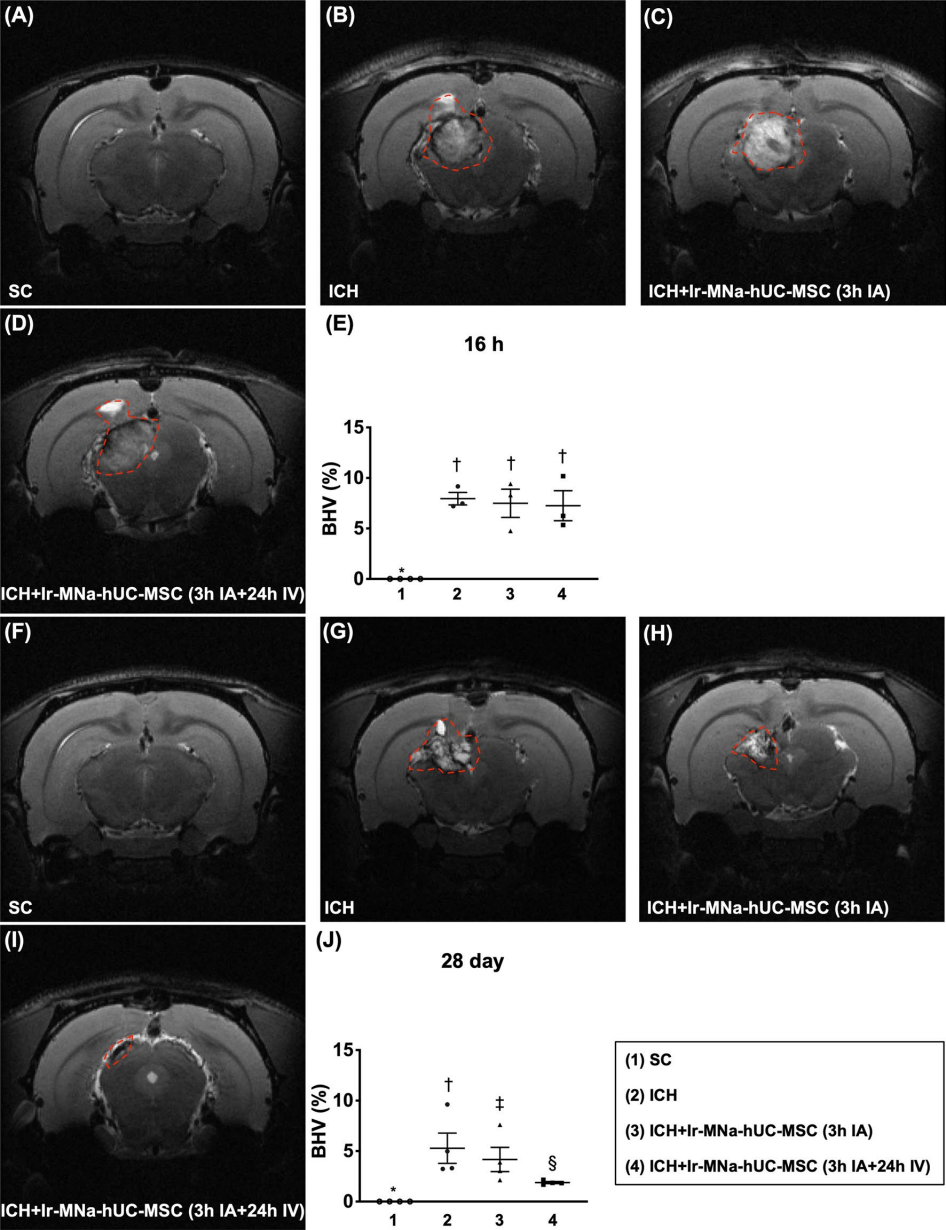
The brain MRI findings for assessment of the brain hemorrhagic volume (BHV) by 16 h and day 28 after intracranial hemorrhage (ICH) induction procedure. **A**–**D** Illustrating the brain MRI finding by 16 h after ICH induction (red dotted line area). **E** Analytical result of BHV, *versus †, *p* < 0.001. **F**–**I** Illustrating the brain MRI finding by day 28 after ICH induction (red dotted line area). **J** Analytical result of BHV, *versus other groups with different symbols (†, ‡, §), *p* < 0.001. All statistical analyses were performed by one-way ANOVA, followed by Bonferroni multiple comparison post hoc test (*n* = 4 for each group). Symbols [(*, †, ‡, §) indicate significance (at 0.05 level). IA = intra-carotid arterial administration; IV intravenous (i.e., tail vein) administration; SC = sham control; ICH = intracranial hemorrhage; Ir-MNa-hUC-MSC = ironic magnetic nanoparticles/human umbilical cord-derived mesenchymal stem cells; MRI = magnetic resonance imaging

To examine the therapeutic effect of hUC-MSCs on reduction of BHV, the brain MRI was utilized in the present study. As our expected, by 16 h after ICH, the BHV was significantly increased in groups 2 to 4 than in group 1, but it showed no difference among the former three groups (Fig. 3). However, by day 28 after ICH induction, the BHV was lowest in group 1, highest in group 2 and significantly higher in group 3 than in group 4, implicating two doses of hUC-MSCs therapy might be better than the one dose counterpart for reducing the brain architectural damage after ICH induction (Fig. 3).

### The hUC-MSCs treatment effectively suppressed inflammatory reaction and generation of oxidative stress in brain by day 28 after ICH induction (Fig. 4)

The protein expressions of TNF-α, MMP-9, IL-1ß, and iNOS, four indicators of inflammatory reaction, were lowest in group 1, highest in group 2 and significantly higher in group 3 than in group 4. Additionally, the protein expressions of NOX-1, NOX-2, and oxidized protein, three indices of oxidative stress, displayed an identical pattern of inflammation among the four groups. On the other hand, the protein expression of IL-10, an indicator of anti-inflammation, exhibited an opposite pattern of inflammation among the groups.

**Fig. 4:**
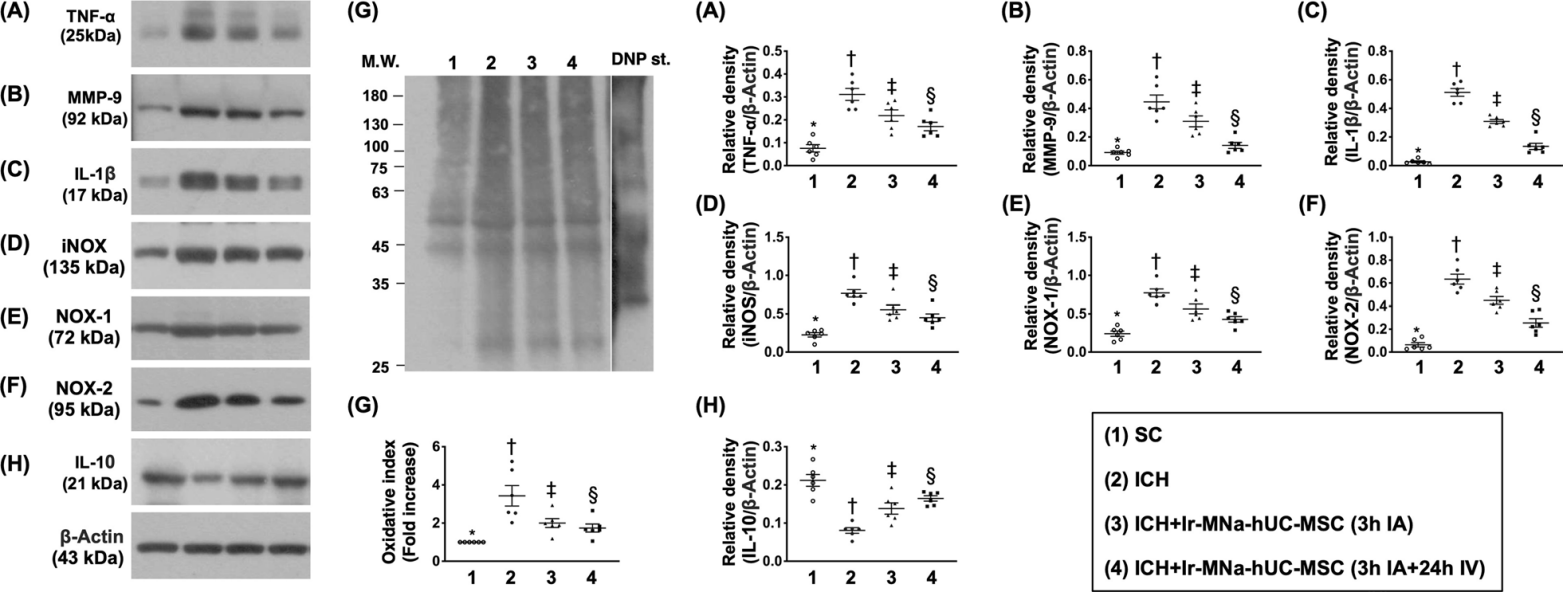
The hUC-MSCs treatment effectively suppressed inflammatory reaction and generation of oxidative stress in brain by day 28 after ICH induction. **A** Protein expression of tumor necrosis factor (TNF)-α, *versus other groups with different symbols (†, ‡, §), *p* < 0.0001. **B** Protein expression of matrix metalloproteinase (MMP)-9, *versus other groups with different symbols (†, ‡, §), *p* < 0.0001. **C** Protein expression of interleukin (IL)-1ß, *versus other groups with different symbols (†, ‡, §), *p* < 0.0001. **D** Protein expression of inducible nitric oxide synthase (iNOS), *versus other groups with different symbols (†, ‡, §), *p* < 0.0001. **E** Protein expression of NOX-1, *versus other groups with different symbols (†, ‡, §), *p* < 0.0001. **F** Protein expression of NOX-2, *versus other groups with different symbols (†, ‡, §), *p* < 0.0001.** G** The oxidized protein expression, *versus other groups with different symbols (†, ‡, §), *p* < 0.0001 (Note: the left and right lanes shown on the upper panel represent protein molecular weight marker and control oxidized molecular protein standard, respectively). M.W. = molecular weight; DNP = 1–3 dinitrophenylhydrazone. **H** Protein expression of interleukin (IL)-10, *versus other groups with different symbols (†, ‡, §), *p* < 0.0001. All statistical analyses were performed by one-way ANOVA, followed by Bonferroni multiple comparison post hoc test (*n* = 6 for each group). Symbols [(*, †, ‡, §) indicate significance (at 0.05 level). IA = intra-carotid arterial administration; IV intravenous (i.e., tail vein) administration; SC = sham control; ICH = intracranial hemorrhage; Ir-MNa-hUC-MSC = ironic magnetic nanoparticles/human umbilical cord-derived mesenchymal stem cells

### The hUC-MSCs treatment effectively downregulated apoptosis, fibrosis and generation of oxidative stress as well as notably preserved the mitochondrial integrity in brain by day 28 after ICH induction (Fig. 5)

As expected, the protein expressions of caspase-3, mitochondrial Bax and PARP, three indicators of apoptosis, were lowest in group 1, highest in group 2 and significantly higher in group 3 than in group 4. Additionally, the protein expressions of TGF-ß and Smad3, two indicators of fibrosis, and cytosolic cytochrome C, an indicator of mitochondrial damaged marker, exhibited an identical pattern of apoptosis among the groups.

**Fig. 5:**
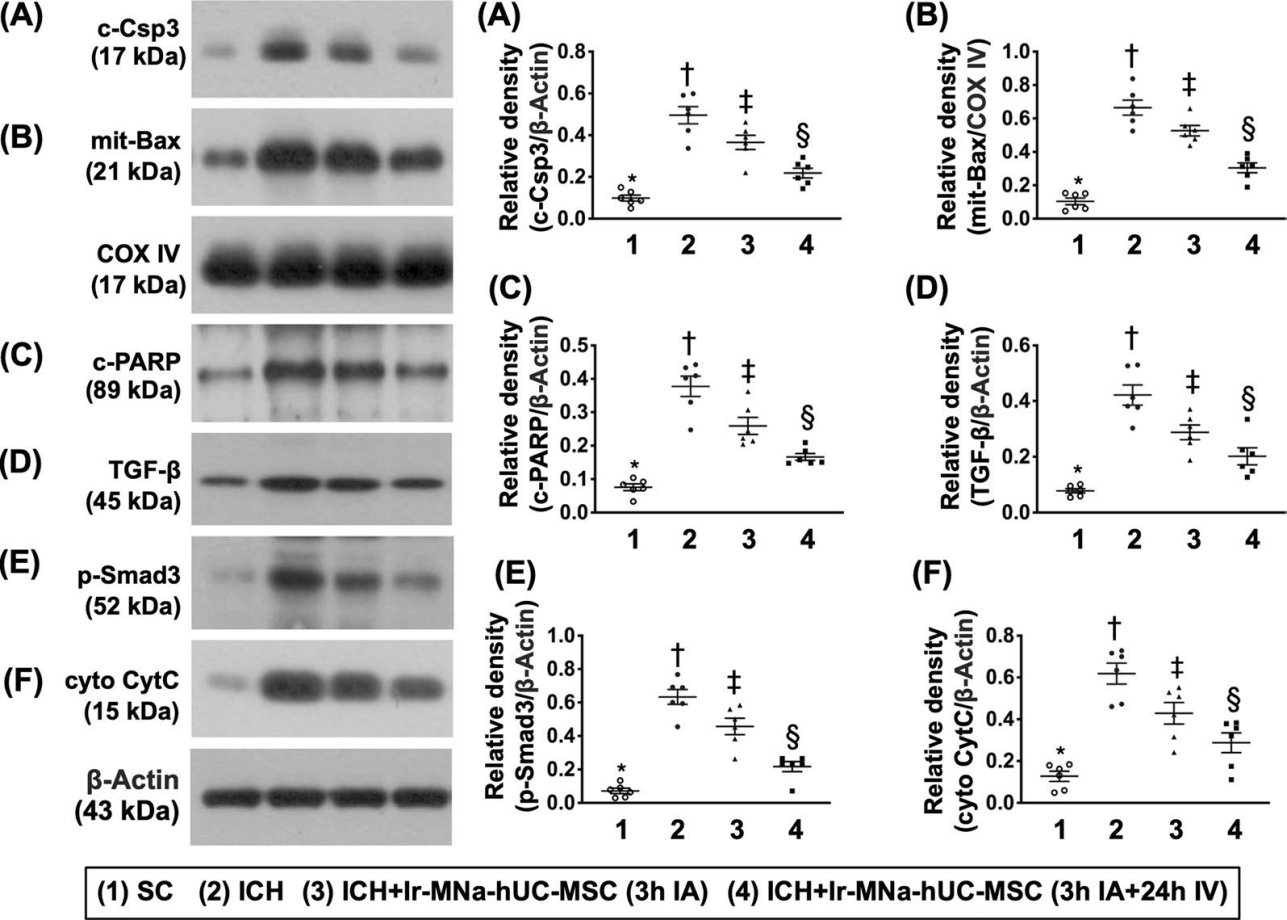
The hUC-MSCs treatment effectively downregulated apoptosis, fibrosis and generation of oxidative stress in brain by day 28 after ICH induction. **A** Protein expressions of caspase-3 (Csp3), *versus other groups with different symbols (†, ‡, §), *p* < 0.0001. **B** Protein expression of mitochondrial (Mit)-Bax, *versus other groups with different symbols (†, ‡, §), *p* < 0.0001. **C** Protein expression of cleaved poly [ADP-ribose] polymerase (c-PARP), *versus other groups with different symbols (†, ‡, §), *p* < 0.0001. **D** Protein expression of transforming growth factor (TGF)-ß, *versus other groups with different symbols (†, ‡, §), *p* < 0.0001. **E** Protein expression of phosphorylated (p)- Smad3, *versus other groups with different symbols (†, ‡, §), *p* < 0.0001. **F** Protein expression of cytosolic cytochrome C (cyto-CytC), *versus other groups with different symbols (†, ‡, §), *p* < 0.0001. All statistical analyses were performed by one-way ANOVA, followed by Bonferroni multiple comparison post hoc test (*n* = 6 for each group). Symbols [(*, †, ‡, §) indicate significance (at 0.05 level). IA = intra-carotid arterial administration; IV intravenous (i.e., tail vein) administration; SC = sham control; ICH = intracranial hemorrhage; Ir-MNa-hUC-MSC = ironic magnetic nanoparticles/human umbilical cord-derived mesenchymal stem cells

### Cellular expressions of inflammation and neuronal structure in brain hemorrhagic zone by day 28 after ICH induction (Figs. 6, 7*)*

**Fig. 6:**
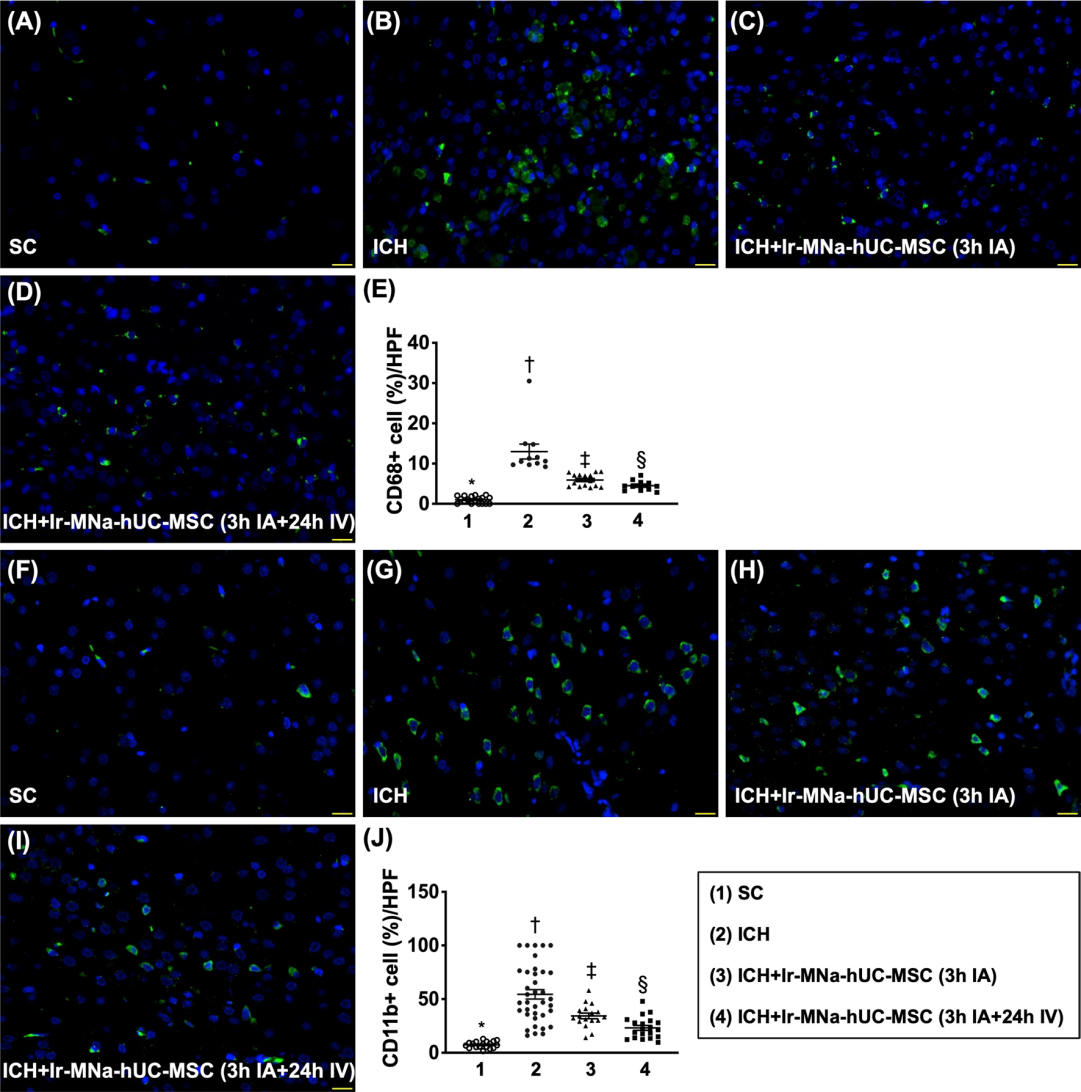
Cellular expressions of inflammation in brain hemorrhagic zone by day 28 after ICH induction. **A**–**D** Illustrating the immunofluorescent (IF) microscopic finding (400 ×) for identification of cellular expression of CD68 (green color). **E** Analytical result of number of CD68+ cells, *versus other groups with different symbols (†, ‡, §), *p* < 0.0001. **F**–**I** Illustrating the IF microscopic finding (400 ×) for identification of cellular expression of CD11b (green color).** J** Analytical result of number of CD11b+ cells, *versus other groups with different symbols (†, ‡, §), *p* < 0.0001. Scale bar in right lower corner represents 20 µm. HPF = high-power field. All statistical analyses were performed by one-way ANOVA, followed by Bonferroni multiple comparison post hoc test (*n* = 6 for each group). Symbols [(*, †, ‡, §) indicate significance (at 0.05 level). IA = intra-carotid arterial administration; IV intravenous (i.e., tail vein) administration; SC = sham control; ICH = intracranial hemorrhage; Ir-MNa-hUC-MSC = ironic magnetic nanoparticles/human umbilical cord-derived mesenchymal stem cells

**Fig. 7:**
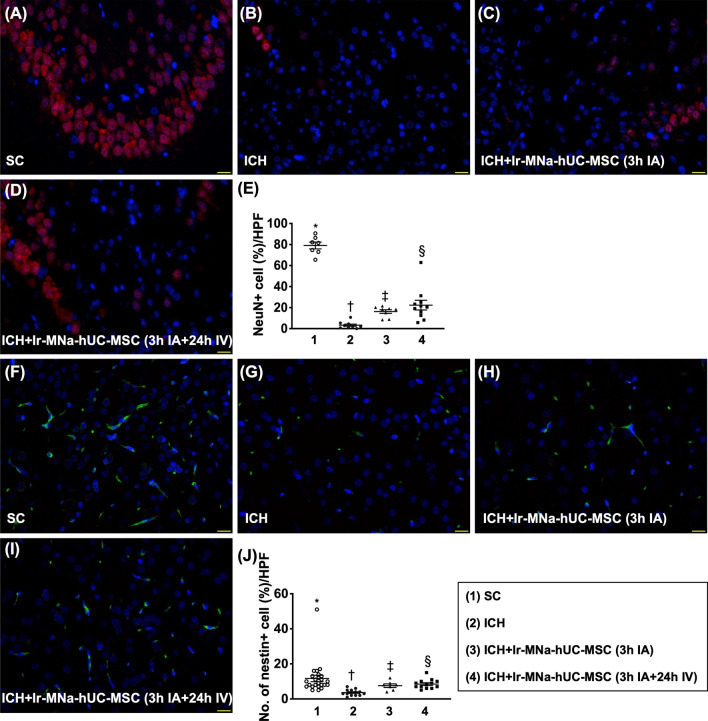
Cellular expressions of neuronal components in brain hemorrhagic zone by day 28 after ICH induction. **A**–**D** Illustrating the immunofluorescent (IF) microscopic finding (400 ×) for identification of the expression of NeuN cells (red color). **E** Analytical result of number of NeuN + cells, *versus other groups with different symbols (†, ‡, §), *p* < 0.0001. **F**–**I** Illustrating the IF microscopic finding (400 ×) for identification of the cellular expression of Nestin (green color). **J** Analytical result of number of Nestin + cells, *versus other groups with different symbols (†, ‡, §), *p* < 0.0001. Scale bar in right lower corner represents 20 µm. HPF = high-power field. All statistical analyses were performed by one-way ANOVA, followed by Bonferroni multiple comparison post hoc test (*n* = 6 for each group). Symbols [(*, †, ‡, §) indicate significance (at 0.05 level). IA = intra-carotid arterial administration; IV intravenous (i.e., tail vein) administration; SC = sham control; ICH = intracranial hemorrhage; Ir-MNa-hUC-MSC = ironic magnetic nanoparticles/human umbilical cord-derived mesenchymal stem cells

When we looked into the IF microscopic finding, we found that the cellular expressions of CD68 and CD11b, two indicators of inflammation in brain hemorrhagic zone, were lowest in group 1, highest in group 2 and significantly higher in group 3 than in group 4 (Fig. 6). Additionally, positively stained NeuN cells, an indicator of neurons, and positively stained Nestin, an indicator of type VI intermediate filament of axon, exhibited an opposite pattern of inflammation among the four groups (Fig. 7).

### Brain edema and neuronal precursor cell biomarkers in brain hemorrhagic zone by day 28 after ICH induction (Fig. 8)

The expression of positively stained doublecortin cells, a microtubule-associated protein expressed by neuronal precursor cells and immature neurons, was highest in group 1, lowest in group 2 and significantly higher in group 4 than in group 3. Additionally, the expression of positively stained AQP4 cells, an indicator of brain edema, exhibited an opposite pattern of doublecortin among the four groups.

**Fig. 8:**
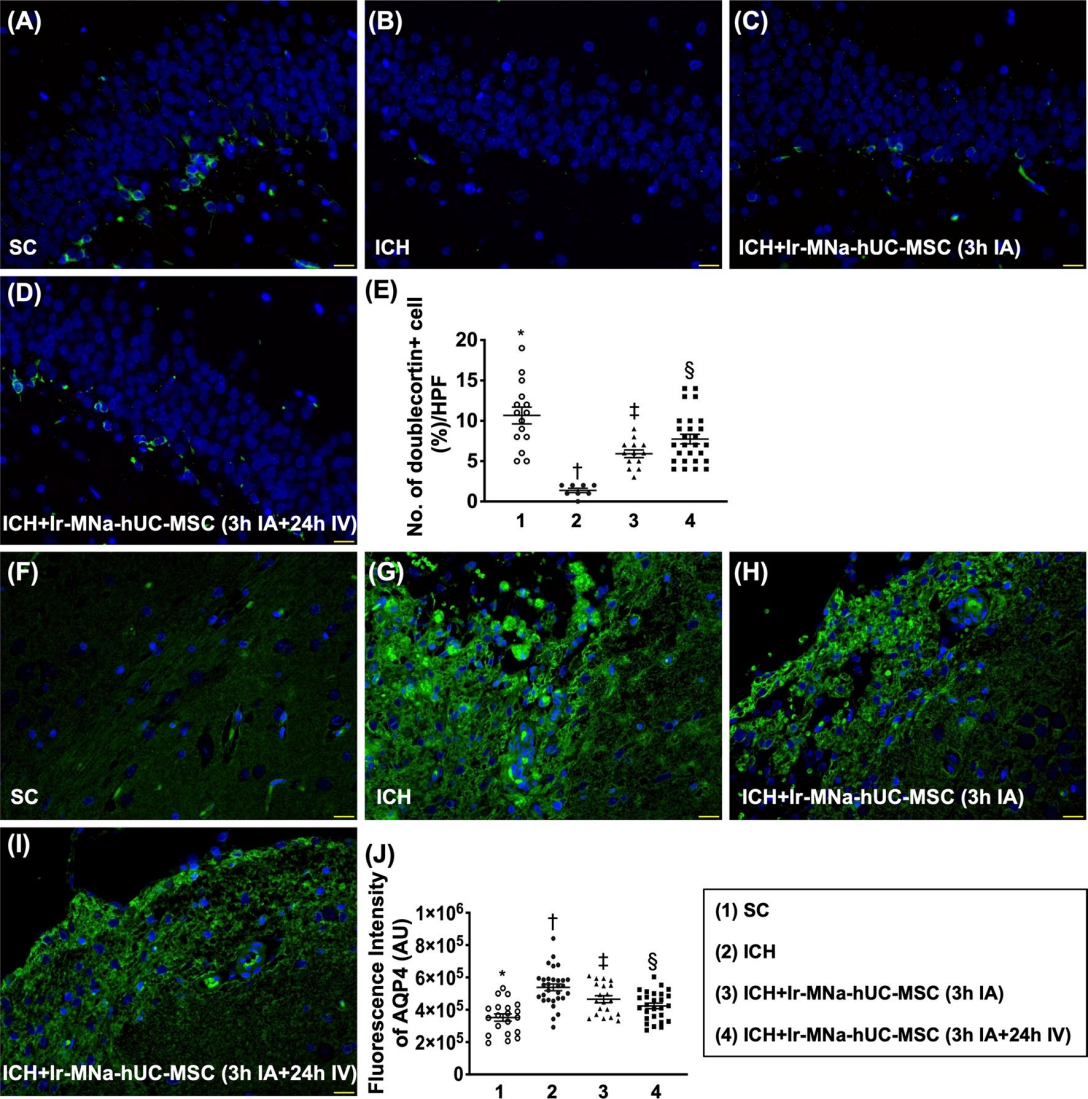
Brain edema and neuronal precursor cell biomarkers in brain hemorrhagic zone by day 28 after ICH induction. **A**–**D** Illustrating the immunofluorescent (IF) microscopic finding (400 ×) for identification of the expression of doublecortin cells (green color).** E** Analytical result of number of doublecortin + cells, *versus other groups with different symbols (†, ‡, §), *p* < 0.0001. **F**–**I** Illustrating the IF microscopic finding (400 ×) for identification of the cellular expression of aquaporin-4 (AQP4) (green color). **J** Analytical result of number of AQP4+ cells, *versus other groups with different symbols (†, ‡, §), *p* < 0.0001. Scale bar in right lower corner represents 20 µm. HPF = high-power field. All statistical analyses were performed by one-way ANOVA, followed by Bonferroni multiple comparison post hoc test (*n* = 6 for each group). Symbols [(*, †, ‡, §) indicate significance (at 0.05 level). IA = intra-carotid arterial administration; IV intravenous (i.e., tail vein) administration; SC = sham control; ICH = intracranial hemorrhage; Ir-MNa-hUC-MSC = Ironic Magnetic Nanoparticles/human umbilical cord-derived mesenchymal stem cells

### Time courses of circulatory, inflammatory and apoptotic biomarkers (Fig. 9)

The flow cytometric analysis demonstrated that the circulatory levels of Ly6G+, MPO+ and CD11b/c+ cells, three inflammatory biomarkers, were highest in group 2, lowest in group 1 and significantly higher in group 3 than in group 4, whereas the early (i.e., AN-V+/PI− cells) and late (i.e., AN-V+/PI+ cells) apoptosis displayed an identical pattern of inflammatory biomarkers among the four groups by day 28 after acute ICH procedure.

**Fig. 9:**
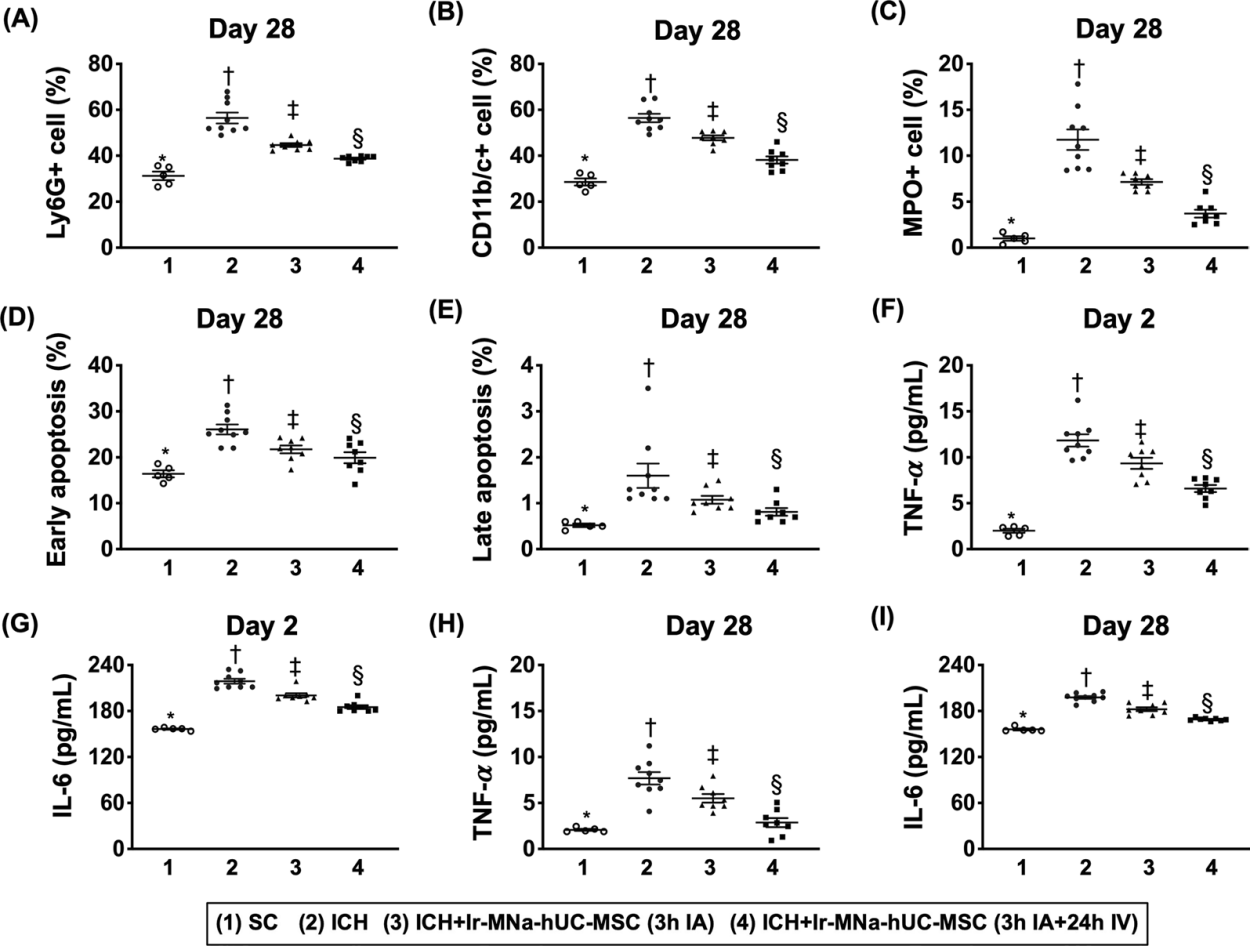
Time courses of circulatory, inflammatory and apoptotic biomarkers. **A** Analytical result of flow cytometric analysis of circulatory level of Ly6G+ cells by day 28 after ICH induction, *versus other groups with different symbols (†, ‡, §), *p* < 0.0001. **B** Analytical result of flow cytometric analysis of circulatory level of CD11b/c+ cells by day 28 after ICH induction, *versus other groups with different symbols (†, ‡, §), *p* < 0.0001. **C** Analytical result of flow cytometric analysis of circulatory level of myeloperoxidase (MPO) + cells by day 28 after ICH induction, *versus other groups with different symbols (†, ‡, §), *p* < 0.0001. **D** Analytical result of flow cytometric analysis of circulatory level of early (AN-V+ /PI− cells) apoptosis of mononuclear cells, *versus other groups with different symbols (†, ‡, §), *p* < 0.0001. **E** Analytical result of flow cytometric analysis of circulatory level of late (i.e., AN-V+ /PI+ cells) apoptosis of mononuclear cells, *versus other groups with different symbols (†, ‡, §), *p* < 0.0001.** F** ELISA showed the circulating level of tumor necrosis factor (TNF)-α by day 2, *versus other groups with different symbols (†, ‡, §), *p* < 0.0001.** G** ELISA showed the circulating level of interleukin (IL)-6 by day 2, *versus other groups with different symbols (†, ‡, §), *p* < 0.0001. **H** ELISA showed the circulating level of TNF-α by day 28, *versus other groups with different symbols (†, ‡, §), *p* < 0.0001.** I** ELISA showed the circulating level of IL-6 by day 28, *versus other groups with different symbols (†, ‡, §), *p* < 0.0001. All statistical analyses were performed by one-way ANOVA, followed by Bonferroni multiple comparison post hoc test (*n* = 6 for each group). Symbols [(*, †, ‡, §) indicate significance (at 0.05 level). IA = intra-carotid arterial administration; IV intravenous (i.e., tail vein) administration; SC = sham control; ICH = intracranial hemorrhage; Ir-MNa-hUC-MSC = ironic magnetic nanoparticles/human umbilical cord-derived mesenchymal stem cells

Furthermore, the ELISA examination demonstrated that the circulatory levels of IL-6 and TNF-α, two indicators of soluble proinflammatory cytokines, exhibited an identical pattern of apoptosis by days 2 and 28 after ICH induction.

## Discussion

Our previous study has demonstrated that autologous adipose-derived mesenchymal stem cells (ADMSCs) [28] and xenogeneic ADMSCs [27] therapies effectively reduced brain infarct size and improved the neurological function in rat after acute IS. Of importance was that there was no immunologic rejection [28] after xenogeneic ADMSC therapy. The most important finding in the present study was that as compared with ICH animals, the BHV was significantly reduced in those of animals after receiving the hUC-MSCs treatment. Additionally, the neurological function was significantly impaired in ICH animals than in those control subjects that were substantially reversed in ICH group after receiving the hUC-MSCs therapy. In this way, our findings were consistent with findings of the previous studies [27, 28].

Interestingly, our previous study has identified that two consecutive doses of allogenic ADMSCs were better than that of merely one dose on attenuating brain death-caused remote organ injury and acute heart-transplant immune rejection [30]. Additionally, our study recently illustrated that two consecutive doses of endothelial progenitor cells were superior to just one dose at preserving left ventricular function in setting of ischemia–reperfusion procedure in rodent [31]. Intriguingly, in a Controlled Randomized Phase I/II Trial, Matas et al. also demonstrated that repeated hUC-MSC dosing was superior to just a single hUC-MSC dose and to hyaluronic acid for treatment of knee osteoarthritis [32].

In the present study, we also found that two consecutive doses of xenogeneic hUC-MSCs were superior to only one dose of this cell therapy for preserving the integrity of brain architecture and neurologic function. In this way, the results of the present study were comparable with the findings of our previous studies [30, 31].

Numerous studies have revealed that tissue/organ in circumstances of ischemia and necrosis always drew forth of large amount of inflammation and oxidative stress, which in turn, stimulated cell stress signaling, resulting in DNA/mitochondrial damage and cell apoptosis and death [25–27, 30, 31, 33, 34]. An essential finding in the present study was that the inflammation and circulation and oxidative stress biomarkers in the brain tissue were found to be substantially increased, followed by remarkably increased in the apoptotic, fibrotic, DNA-damaged and mitochondrial damage biomarkers in hemorrhagic brain tissues. In this way, our findings, in addition to corroborating with the findings of the previous studies [25–27, 30, 31, 33, 34], could, at least in part, explain why the BHV was markedly increased in ICH animals. Paramount importance was that these molecular-cellular perturbations were notably reversed by one dose and more notably reversed by two consecutive doses of cell therapy.

Intriguingly, to successfully track the stem cells homing from circulation into kidney parenchyma using MRI in acute kidney ischemia–reperfusion injury of living rodent has been reported by our recent study [35]. However, to the best of our knowledge, no tracking of stem cell homing into the brain either from intra-arterial or intravenous transfusion in living animals by brain MRI was reported. Although one recent study [36] from Kang et al. also demonstrated that delivery of iron oxide nanoparticle-coated in human embryonic stem cell-derived spherical neural masses for treatment of ICH in rat could be clearly identified in the brain tissue, the identification of iron oxide nanoparticle-coated stem cells in the brain was only by the histopathological examination rather than by brain MRI in living animals. One interesting finding in the present study was that the brain MRI examination could identified the Ir-MNa-coated hUC-MSCs in the ischemic area of living animals by 4 h after ICH, suggesting a rapid mobilization of stem cells from circulation into brain damage area could be, at least in part, due to the effect of magnet over the rat head during cell therapy.

Based on the results of the present study, we proposed that our findings contained several novelties. First, to put a magnet to the head might quickly attract stem cells into the ICH region for tissue/neuronal regenerations. This finding would provide an essential information for the future clinical practice for purpose of more administered stem cell homing into the patient’s organs/tissues, including the brain for tissue regeneration. Second, the study did not find any side effect or immune rejection phenomenon (i.e., enough safety) in ICH rats, highlighting that xenogeneic MSCs also have the property of immune privilege even for two-dose strategic management, and therefore, suggesting that allogenic MSCs therapy for the patients could be safe. Third, we developed a novel method of utilizing non-invasive brain MRI for living animals could easily track the destination of transfused Ir-MNa-coated hUC-MSCs in rat after ICH, suggesting that this method could be utilized in our future daily clinical practice. Fourth, two consecutive doses of cell therapy were better than single dose counterpart for improving the neurological function and preserving the integrity of brain architecture, suggesting that two doses of therapeutic regimen should be considered for the clinical application for those patients who will receive cell therapy. Finally, of importance was that there was no tumorigenesis to be found by brain MRI and histopathological study within a study period of 28 days.

## Study limitation

**Fig. 10:**
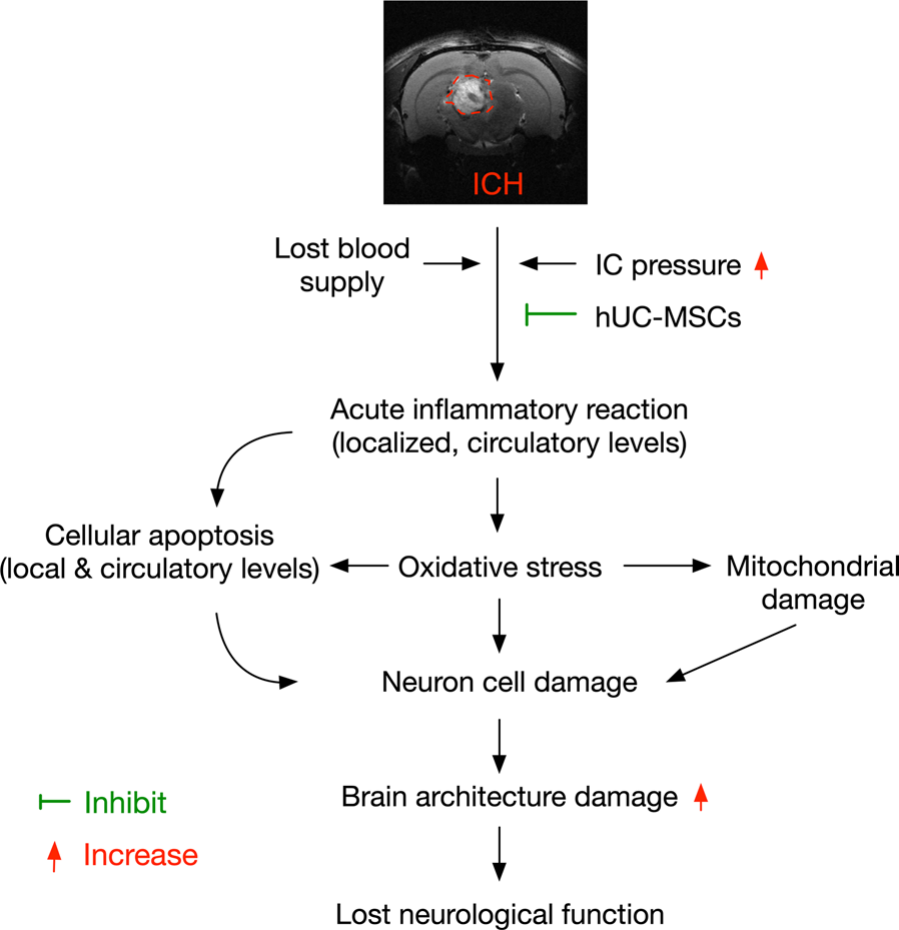
Illustrating the Schematically proposed mechanism of hUC-MSCs therapy for protecting the brain against ICH damage. ICH = intracranial hemorrhage; hUC-MSCs = human umbilical cord-derived mesenchymal stem cells

Our study has limitations. First, the study period was relative short (i.e., only 28 days) that could not provide information regarding the long-term impact of hUC-MSCs on neurological outcome regarding the presence or absence of tumorigenesis. Second, without stepwise increase in dosage of hUC-MSCs, we did not know which dosage of this cell therapy was optimal for ICH. Third, although extensive works have been done by the present study, the exactly underlying mechanism for UC-MSCs treatment on protecting neurological outcomes in rat after ICH is currently unclearly. Thus, we could only be based on our findings to provide a schematically proposed mechanism of hUC-MSCs treatment on protecting the neurological function and brain architectural integrity in rat after ICH (Fig. 10).

## Conclusions

In conclusion, consecutive administration of two doses of hUC-MSCs might be better than merely one dose counterpart for protecting the brain and neurological function from ICH-induced damage. The Ir-MNa-coated hUC-MSCs can serve as a suitable tool for tracking hUC-MSCs to the destination.

## Supplementary Information

**Additional file 1.**: Supplementary figure S1. Schematically illustrated the experimental flow chart of the present study. ICH = intracranial hemorrhage; hUC-MSCs = human umbilical cord-derived mesenchymal stem cells; MRI = magnetic resonance imaging.

## Data Availability

The datasets of present study can be available from the corresponding author upon reasonable request.

## References

[1] Sacco RL, Kasner SE, Broderick JP, Caplan LR, Connors JJ, Culebras A, Elkind MS, George MG, Hamdan AD, Higashida RT (2013). An updated definition of stroke for the 21st century: a statement for healthcare professionals from the American Heart Association/American Stroke Association. Stroke.

[2] Donnan GA, Fisher M, Macleod M, Davis SM (2008). Stroke. Lancet.

[3] Hu HH, Sheng WY, Chu FL, Lan CF, Chiang BN (1992). Incidence of stroke in Taiwan. Stroke.

[4] Andersen KK, Olsen TS, Dehlendorff C, Kammersgaard LP (2009). Hemorrhagic and ischemic strokes compared: stroke severity, mortality, and risk factors. Stroke.

[5] Broderick JP, Brott T, Tomsick T, Miller R, Huster G (1993). Intracerebral hemorrhage more than twice as common as subarachnoid hemorrhage. J Neurosurg.

[6] Aguilar MI, Brott TG (2011). Update in intracerebral hemorrhage. Neurohospitalist.

[7] Di Carlo A (2009). Human and economic burden of stroke. Age Ageing.

[8] Masdeu JC, Rubino FA (1984). Management of lobar intracerebral hemorrhage: medical or surgical. Neurology.

[9] Morotti A, Goldstein JN (2016). Diagnosis and management of acute intracerebral hemorrhage. Emerg Med Clin N Am.

[10] Kim JY, Bae HJ (2017). Spontaneous intracerebral hemorrhage: management. J Stroke.

[11] Al-Kawaz MN, Hanley DF, Ziai W (2020). Advances in therapeutic approaches for spontaneous intracerebral hemorrhage. Neurotherapeutics.

[12] Haupenthal D, Kuramatsu JB, Volbers B, Sembill JA, Mrochen A, Balk S, Hoelter P, Lucking H, Engelhorn T, Dorfler A (2021). Disability-adjusted life-years associated with intracerebral hemorrhage and secondary injury. JAMA Netw Open.

[13] Steiner T, Vincent C, Morris S, Davis S, Vallejo-Torres L, Christensen MC (2011). Neurosurgical outcomes after intracerebral hemorrhage: results of the Factor Seven for Acute Hemorrhagic Stroke Trial (FAST). J Stroke Cerebrovasc Dis.

[14] Testai FD, Aiyagari V. Acute hemorrhagic stroke pathophysiology and medical interventions: blood pressure control, management of anticoagulant-associated brain hemorrhage and general management principles. Neurol Clin. 2008;26:963–85, viii-ix. 10.1016/j.ncl.2008.06.001 19026899

[15] Engler AJ, Sen S, Sweeney HL, Discher DE (2006). Matrix elasticity directs stem cell lineage specification. Cell.

[16] Ryan JM, Barry FP, Murphy JM, Mahon BP (2005). Mesenchymal stem cells avoid allogeneic rejection. J Inflamm (Lond).

[17] Jiang Y, Jahagirdar BN, Reinhardt RL, Schwartz RE, Keene CD, Ortiz-Gonzalez XR, Reyes M, Lenvik T, Lund T, Blackstad M (2002). Pluripotency of mesenchymal stem cells derived from adult marrow. Nature.

[18] Franco Lambert AP, Fraga Zandonai A, Bonatto D, Cantarelli Machado D, Pegas Henriques JA (2009). Differentiation of human adipose-derived adult stem cells into neuronal tissue: does it work?. Differentiation.

[19] Figueroa FE, Carrion F, Villanueva S, Khoury M (2012). Mesenchymal stem cell treatment for autoimmune diseases: a critical review. Biol Res.

[20] Sun L, Wang D, Liang J, Zhang H, Feng X, Wang H, Hua B, Liu B, Ye S, Hu X (2010). Umbilical cord mesenchymal stem cell transplantation in severe and refractory systemic lupus erythematosus. Arthritis Rheum.

[21] Chao YH, Tsai C, Peng CT, Wu HP, Chan CK, Weng T, Wu KH (2011). Cotransplantation of umbilical cord MSCs to enhance engraftment of hematopoietic stem cells in patients with severe aplastic anemia. Bone Marrow Transpl.

[22] Wu KH, Sheu JN, Wu HP, Tsai C, Sieber M, Peng CT, Chao YH (2013). Cotransplantation of umbilical cord-derived mesenchymal stem cells promote hematopoietic engraftment in cord blood transplantation: a pilot study. Transplantation.

[23] Li Y, Hu G, Cheng Q (2015). Implantation of human umbilical cord mesenchymal stem cells for ischemic stroke: perspectives and challenges. Front Med.

[24] Lee HS, Kim KS, Lim HS, Choi M, Kim HK, Ahn HY, Shin JC, Joe YA (2015). Priming Wharton's jelly-derived mesenchymal stromal/stem cells with ROCK inhibitor improves recovery in an intracerebral hemorrhage model. J Cell Biochem.

[25] Yip HK, Lin KC, Sung PH, Chiang JY, Yin TC, Wu RW, Chen KH (2021). Umbilical cord-derived MSC and hyperbaric oxygen therapy effectively protected the brain in rat after acute intracerebral haemorrhage. J Cell Mol Med.

[26] Chen KH, Shao PL, Li YC, Chiang JY, Sung PH, Chien HW, Shih FY, Lee MS, Chen WF, Yip HK (2020). Human umbilical cord-derived mesenchymal stem cell therapy effectively protected the brain architecture and neurological function in rat after acute traumatic brain injury. Cell Transpl.

[27] Chen KH, Chen CH, Wallace CG, Yuen CM, Kao GS, Chen YL, Shao PL, Chen YL, Chai HT, Lin KC (2016). Intravenous administration of xenogenic adipose-derived mesenchymal stem cells (ADMSC) and ADMSC-derived exosomes markedly reduced brain infarct volume and preserved neurological function in rat after acute ischemic stroke. Oncotarget.

[28] Leu S, Lin YC, Yuen CM, Yen CH, Kao YH, Sun CK, Yip HK (2010). Adipose-derived mesenchymal stem cells markedly attenuate brain infarct size and improve neurological function in rats. J Transl Med.

[29] Chen YL, Tsai TH, Wallace CG, Chen YL, Huang TH, Sung PH, Yuen CM, Sun CK, Lin KC, Chai HT (2015). Intra-carotid arterial administration of autologous peripheral blood-derived endothelial progenitor cells improves acute ischemic stroke neurological outcomes in rats. Int J Cardiol.

[30] Yip HK, Lee MS, Sun CK, Chen KH, Chai HT, Sung PH, Lin KC, Ko SF, Yuen CM, Liu CF (2017). Therapeutic effects of adipose-derived mesenchymal stem cells against brain death-induced remote organ damage and post-heart transplant acute rejection. Oncotarget.

[31] Yeh JN, Yang RR, Wallace CG, Huang CR, Chu YC, Yip HK, Guo J (2021). Impact of one versus two consecutive doses of endothelial cells (EPCs) and EPCs-derived condition medium on protecting myocardium from acute ischemia-reperfusion injury in rat. Cell Transpl.

[32] Matas J, Orrego M, Amenabar D, Infante C, Tapia-Limonchi R, Cadiz MI, Alcayaga-Miranda F, Gonzalez PL, Muse E, Khoury M (2019). Umbilical cord-derived mesenchymal stromal cells (MSCs) for knee osteoarthritis: repeated MSC dosing is superior to a single MSC dose and to hyaluronic acid in a controlled randomized phase I/II trial. Stem Cells Transl Med.

[33] Yang CH, Sheu JJ, Tsai TH, Chua S, Chang LT, Chang HW, Lee FY, Chen YL, Chung SY, Sun CK (2013). Effect of tacrolimus on myocardial infarction is associated with inflammation, ROS, MAP kinase and Akt pathways in mini-pigs. J Atheroscler Thromb.

[34] Yeh JN, Sung PH, Chiang JY, Sheu JJ, Huang CR, Chu YC, Chua S, Yip HK (2021). Early treatment with combination of SS31 and entresto effectively preserved the heart function in doxorubicin-induced dilated cardiomyopathic rat. Biomed Pharmacother.

[35] Sheu JJ, Sung PH, Wallace CG, Yang CC, Chen KH, Shao PL, Chu YC, Huang CR, Chen YL, Ko SF (2020). Intravenous administration of iPS-MSC(SPIONs) mobilized into CKD parenchyma and effectively preserved residual renal function in CKD rat. J Cell Mol Med.

[36] Kang MK, Kim TJ, Kim YJ, Kang L, Kim J, Lee N, Hyeon T, Lim MS, Mo HJ, Shin JH (2020). Targeted delivery of iron oxide nanoparticle-loaded human embryonic stem cell-derived spherical neural masses for treating intracerebral hemorrhage. Int J Mol Sci..

