# Supplementary figures and images for: Dose-dependent benefits of iron-magnetic nanoparticle-coated human umbilical-derived mesenchymal stem cell treatment in rat intracranial hemorrhage model
Source: Stem Cell Res Ther. 2022 Jun 21;13:265. doi: 10.1186/s13287-022-02939-4 (PMC9210819; doi:10.1186/s13287-022-02939-4)

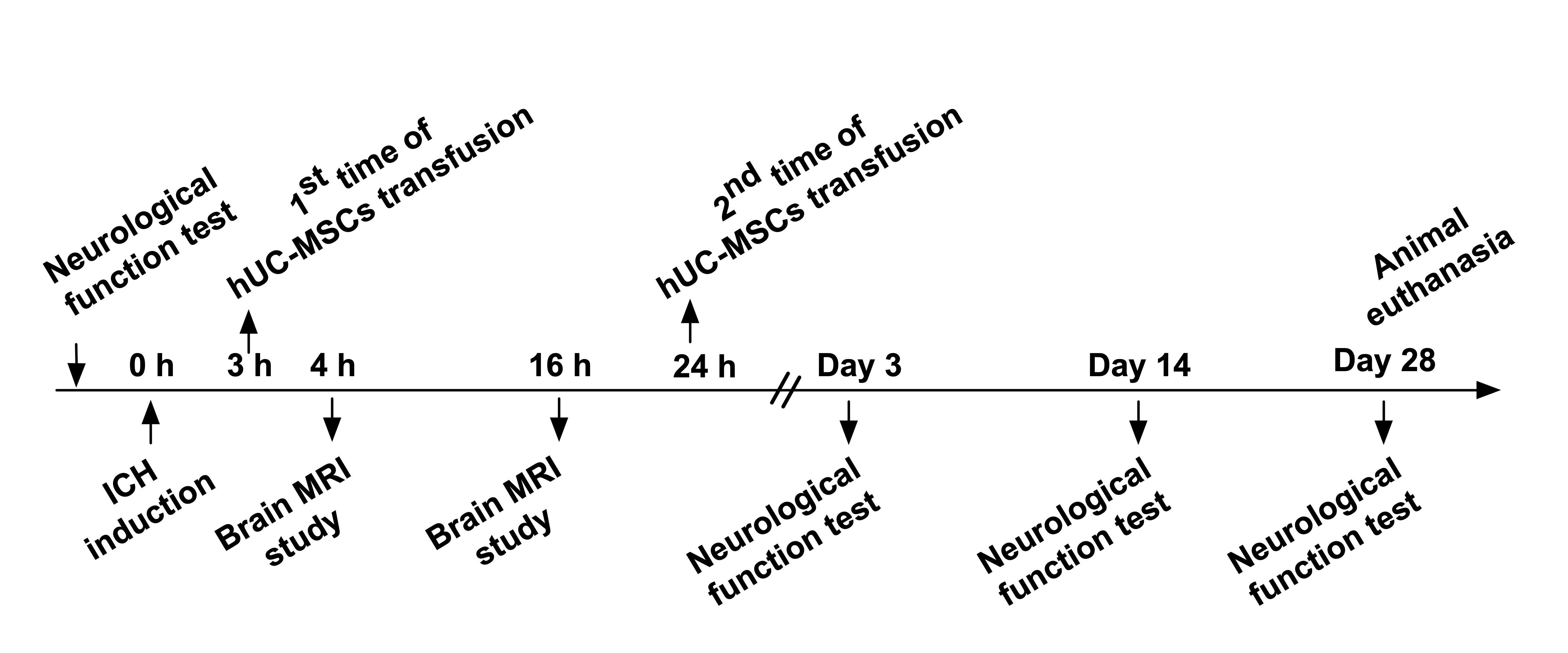

Supplement: Supplementary file 1 — Additional file 1.: Supplementary figure S1. Schematically illustrated the experimental flow chart of the present study. ICH = intracranial hemorrhage; hUC-MSCs = human umbilical cord-derived mesenchymal stem cells; MRI = magnetic resonance imaging. [file 13287_2022_2939_MOESM1_ESM.jpg]
